# Inverse Design of Photonic Surfaces via High throughput Femtosecond Laser Processing and Tandem Neural Networks

**DOI:** 10.1002/advs.202401951

**Published:** 2024-04-29

**Authors:** Minok Park, Luka Grbčić, Parham Motameni, Spencer Song, Alok Singh, Dante Malagrino, Mahmoud Elzouka, Puya H. Vahabi, Alberto Todeschini, Wibe Albert de Jong, Ravi Prasher, Vassilia Zorba, Sean D. Lubner

**Affiliations:** ^1^ Energy Technologies Area Lawrence Berkeley National Laboratory Berkeley CA 94720 USA; ^2^ Applied Mathematics and Computational Research Division Lawrence Berkeley National Laboratory Berkeley CA 94720 USA; ^3^ School of Information University of California at Berkeley Berkeley CA 94709 USA; ^4^ School of Computer Science & Information Technology Lucerne University of Applied Sciences and Arts Lucerne 6343 Switzerland; ^5^ Department of Mechanical Engineering University of California at Berkeley Berkeley CA 94709 USA; ^6^ Department of Mechanical Engineering, Division of Materials Science and Engineering Boston University Boston MA 02215 USA

**Keywords:** deep learning, femtosecond laser processing, inverse design, machine learning, photonic surface, tandem neural network

## Abstract

This work demonstrates a method to design photonic surfaces by combining femtosecond laser processing with the inverse design capabilities of tandem neural networks that directly link laser fabrication parameters to their resulting textured substrate optical properties. High throughput fabrication and characterization platforms are developed that generate a dataset comprising 35280 unique microtextured surfaces on stainless steel with corresponding measured spectral emissivities. The trained model utilizes the nonlinear one‐to‐many mapping between spectral emissivity and laser parameters. Consequently, it generates predominantly novel designs, which reproduce the full range of spectral emissivities (average root‐mean‐squared‐error < 2.5%) using only a compact region of laser parameter space 25 times smaller than what is represented in the training data. Finally, the inverse design model is experimentally validated on a thermophotovoltaic emitter design application. By synergizing laser‐matter interactions with neural network capabilities, the approach offers insights into accelerating the discovery of photonic surfaces, advancing energy harvesting technologies.

## Graphical abstract

By integrating high throughput femtosecond laser processing with a tandem neural network framework, the inverse design of photonic surfaces is showcased with tailored optical properties. The trained model adeptly utilizes the nonlinear one‐to‐many mapping relationship between spectral emissivity and laser parameters. This approach provides insights into expediting the exploration of photonic surfaces, thereby advancing energy harvesting technologies.

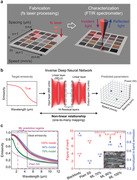

## 1 Introduction

Optical metasurfaces, engineered for specific optical properties, enable manipulation of electromagnetic radiation beyond the capabilities of natural materials^[^ ^1^ ^]^ and have been utilized in diverse applications, including sensing,^[^ ^2^ ^]^ negative refractive indices,^[^ ^3^ ^]^ and spectrally selective light absorbers.^[^ ^4^ ^]^ In particular, controlling light‐matter interactions in the visible to infrared wavelength range is crucial for renewable energy technologies such as parabolic troughs^[^ ^5^ ^]^ and next‐generation concentrated solar power towers,^[^ ^6^ ^]^ passive radiative cooling,^[^ ^7^ ^]^ and thermophotovoltaics (TPV).^[^ ^8^ ^]^ This behavior is largely governed by a material's spectral emissivity, which is the energy radiated from a material's surface at each wavelength normalized to that of a perfect emitter (known as a blackbody), and ranges from 0 to 1.^[^ ^9^ ^]^ Therefore, enhancing system performance requires photonic surfaces with tailored spectral emissivity that selectively controls radiative energy transport, a task that is increasingly being addressed through the application of machine learning (ML).

Specifically, the inverse design of optical metasurfaces,^[^ ^10^ ^]^ such as high efficiency thermal emitter design,^[^ ^11^ ^]^ has been enabled by ML and deep neural network (DNN) models,^[^ ^12^ ^]^ including adversarial autoencoders (AAs),^[^ ^13^ ^]^ generative adversarial networks (GANs),^[^ ^14^ ^]^ and variation autoencoders (VAEs).^[^ ^15^ ^]^ Trained ML models suggest optimized structural parameters in a single step to produce a design that exhibits the target optical properties. This circumvents the need for computationally expensive iterative direct electrodynamic simulations. However, these approaches often fail to adequately handle and make use of one‐to‐many mapping scenarios and nonlinear relationships between the design input and output spaces that are common in real‐world manufacturing.^[^ ^16^ ^]^ This challenge becomes particularly pronounced when training data overly emphasizes specific correlations over others. Furthermore, they frequently overlook the practical constraints of real‐world fabrication, scalability, and uncertainties; they are often entirely computational, lacking experimental validation of design predictions from the trained model, particularly for novel situations. Consequently, the model's capabilities are constrained, limiting its practicality in generating functional devices and deepening understanding of these complex relationships.

Laser ablation, the selective material removal from a surface through interaction with a high power laser, has emerged as a facile technique for precise yet scalable surface morphology alteration and subsequent property enhancement.^[^ ^17^ ^]^ Ultrafast femtosecond (fs) lasers in particular, stand out for their ability to create diverse surface structures ranging from hundreds of nanometers to micrometer scale through self‐organization or direct laser writing techniques.^[^ ^18^ ^]^ These structures can produce a large diversity of optical properties over a broad range of wavelengths, especially in metallic substrates.^[^ ^18^, ^19^ ^]^ The resulting spectral emissivity can change in significant ways when manipulating incident laser parameters such as its power, the speed at which it is rastered over the substrate for a fixed pulse repetition rate, or the precise spacing between consecutive rastered scan lines. Nevertheless, the relationships between laser parameters and corresponding optical properties are complex and difficult to model due to the multiphysics nature of the ablation process.^[^ ^17b,c^ ^]^ Therefore, elucidating the function that directly maps laser parameters to spectral emissivity through a ML approach has the significant potential to enable the inverse design of photonic surfaces by understanding such relationships.

In this work, we demonstrate inverse design of photonic surfaces by developing high throughput fs laser fabrication and optical property characterization platforms combined with a tandem neural network (TNN) framework. We fabricated 35280 distinct surface geometries on stainless steel (SS) substrates and characterized their optical properties to train and test the TNN model, which integrates both forward and inverse DNNs. The trained DNN models serve as a surrogate model to handle the complex one‐to‐many mapping between optical properties and laser fabrication parameters at a level of detail beyond what either direct simulations or optimized experiments can provide. Consequently, the models can produce novel parameter designs not found in the training data and generate the full range of desired optical properties using only a specific, compact region within laser parameter spaces. We experimentally validated the efficacy of the TNN for the design of a spectral filter thermal emitter fabricated out of SS for a lead selenide‐based TPV. Our integrated approach showcases the ability to accelerate the discovery and inverse design of photonic surfaces for various energy harvesting and storage applications.

## 2 High throughput fs Laser Fabrication and Optical Property Characterization

**Figure 1:**
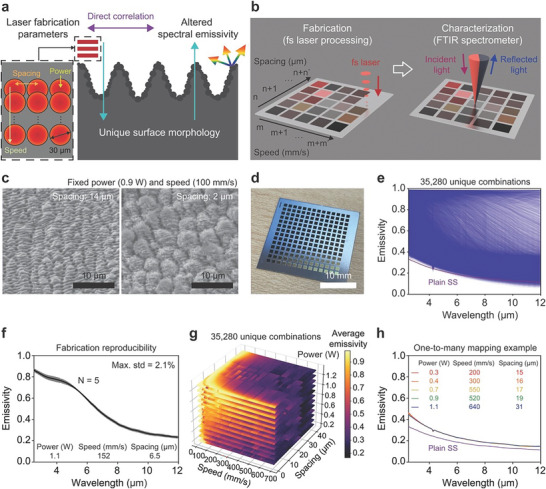
High throughput fs laser fabrication and optical property characterization of photonic surfaces for training data. a) Schematic of three laser parameters (scanning speed, spacing, and power) that govern surface morphology and corresponding spectral emissivity during laser fabrication. Laser spot diameter is 30 µm. b) Layout for automated high throughput fs laser fabrication and optical property characterization using FTIR. c) SEM images of two representative surface morphologies, fabricated using the same laser power (0.9 W) and scanning speed (100 mm s^−1^), but different laser spacings (14 µm left image; 2 µm right image). The black scale bars are 10 µm. d) Example picture of fabricated surfaces on SS. The white scale bar is 10 mm. e) Measured spectral emissivity for all 35280 structures. f) Measured spectral emissivities of 5 different samples independently fabricated using identical laser processing parameters (power: 1.1 W; speed: 152 mm s^−1^; spacing: 6.5 µm). The maximum standard deviation is 2.1%, demonstrating reproducibility. g) Distribution of unweighted average emissivity for all 35280 structures as a function of laser power, spacing, and speed. h) Example of one‐to‐many mapping; 5‐different laser parameter sets produce similar optical properties.

As shown in **Figure** **1**a, the ablation dynamics involving the restructuring and ejection of material via melting and evaporation during fs laser‐material interactions can produce distinct surface morphologies.^[^ ^17^, ^20^ ^]^ The characteristics of these new morphologies are directly influenced by factors including laser power, scanning speed, and spacing. Altered surface morphologies can produce diverse spectral emissivities highly distinct from the original substrate optical properties. Understanding the direct influence of fabrication laser parameters on resulting spectral emissivity can accelerate the attainment of target optical properties by bypassing the need for iterative loops simulating the complicated relationship between surface morphology and corresponding optical characteristics. To construct datasets for the ML models, we employed high throughput fs laser fabrication (< 2 seconds per automated sample fabrication) and custom microscope Fourier Transform Infrared spectrometer (FTIR) optical property characterization, as outlined in Figure 1b (further details in Methods).

Specifically, an ultrafast fs laser (500 fs pulse duration, 1030 nm wavelength, and 30 µm focused beam diameter) was used to produce unique photonic structures, each exhibiting a different spectral emissivity contingent upon distinct laser processing conditions. For instance, altering only the spacing parameter while maintaining other variables constant (0.9 W power and a scanning speed of 100 mm s^−1^) results in more pronounced surface structures with a fine spacing of 2 µm compared to 14 µm, as observed through scanning electron microscopy (SEM) images in Figure 1c. We systematically adjusted laser power (0.2 W to 1.3 W in 0.1 W increments; see Table S1 (Supporting Information) for laser conditions in intensity and fluence), scanning speed (10 mm s^−1^ to 700 mm s^−1^ in 10 mm s^−1^ increments), and line spacing (1 µm to 42 µm in 1 µm increments) to fabricate a total of 35280 unique structures (Figure S1, Supporting Information). To expedite the fabrication process for all 35280 different combinations, each distinct set of parameters within this three‐dimensional parameter space is applied to individual 1 mm^2^ areas on SS substrates (each fabrication process completes in less than a couple of seconds). This approach enables high throughput fabrication and corresponding data generation, as presented in Figure 1d, Video S1 (Supporting Information).

Following the fabrication process we characterized the spectral emissivities of all 35280 surfaces using our customized automated microscope FTIR (Figure 1e; Figure S2, Supporting Information). We targeted the spectral range from 2.5 µm to 12 µm wavelengths. This regime largely aligns with the scope of our intended thermal emission for energy harvesting and storage applications operating at temperatures ranging from 241 K to 1159 K, in accordance with Wien's displacement law. For example, varying the laser power while maintaining a fixed spacing of 16 µm and speed of 100 mm s^−1^ produces systematic modifications to the spectral emissivity (Figure S3, Supporting Information). To ensure the reproducibility of fabricated surfaces and their corresponding properties, duplicate surfaces were generated on 5 different substrates using identical laser parameters (power of 1.1 W, speed of 152 mm s^−1^, and spacing of 6.5 µm), shown in Figure 1f, resulting in a maximum emissivity standard deviation of only 2.1% over the entire observed spectral range.

The unweighted average (gray) emissivities of all 35280 samples are plotted in Figure 1g; Figures S4, and S5 (Supporting Information) as a function of their corresponding location in the laser parameter space. Average emissivity ranges from 0.23 to 0.99 (Figure S4, Supporting Information). Higher scanning speed and higher spacing tend to yield lower average emissivity values, approximating the original substrate's average emissivity of 0.18, due to the utilization of fewer laser pulses to texture the target surface resulting in less light trapping. While the influence of laser power on optical property variation is less pronounced, scanning speed primarily affects average emissivity (i.e., lower scanning speed provides higher spectral emissivity, indicating that more pulses are irradiated on the target surface; Figure S5, Supporting Information). Notably, Figure 1g,h, and Figure S5 (Supporting Information) demonstrate the presence of multiple parameter combinations capable of producing the same average emissivity – an example of the one‐to‐many mapping.

## 3 Architecture and Training of the TNN Framework

**Figure 2:**
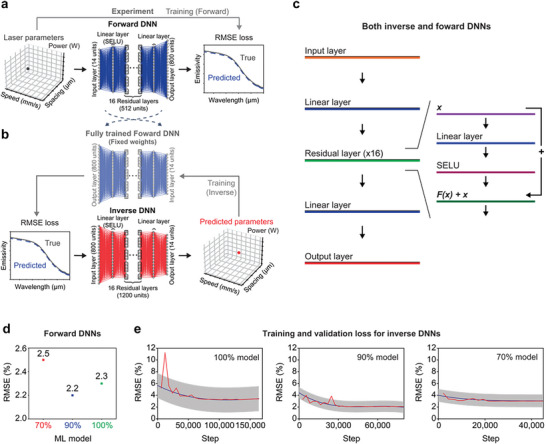
Architecture and training of the TNN framework. a) Forward DNN training, and b) inverse DNN training integrated with fully trained forward DNN. The laser power parameter is one‐hot encoded to maintain its discreteness. The emissivity values are interpolated across 800 linearly distributed wavelengths, ensuring consistent distribution of the spectral emissivity values. c) Structure of each of the 16 residual hidden layers within both the forward and inverse DNN. The input *x* is passed through a linear transformation followed by the SELU activation function (both transformations denoted as *F*). The transformed value, *F(x)*, is then added to the original input *x* to produce the residual layer's output *F(x)+x*. d) RMSE for three forward DNNs; 100% model, 90% model, and 70% model. e) Loss functions associated with the TNN training process. The red line shows the validation loss, the blue line represents the average training loss, and the gray band indicates the standard deviation of the training loss oscillations. The graphs are differentiated into three models: 100% model, 90% model, and 70% model, regarding what fraction of total possible data is used for training.

TNN frameworks combine forward and inverse DNNs and are gaining prominence over alternatives such as AAs, VAEs, and GANs for materials' inverse design.^[^ ^10^, ^16^, ^21^ ^]^ The TNN ensures independent optimization of forward and inverse models for easier training and more precise results. This separation enhances interpretability crucial for the complex nonlinear landscape of laser parameter based photonic surfaces design. **Figure** **2** shows TNN's training procedure and individual components which allow for managing the intricacies of inverse design, mitigating challenges like mode collapse and non‐uniqueness, and supporting a robust mutual loss function for consistent input‐output solutions. More specifically, the one‐to‐many mapping inherent in the inverse design of photonic surfaces (Figure 1h) makes training a stand‐alone DNN for predicting laser parameters challenging because of the difficulty in effectively minimizing the loss function, as elaborated in Figure S6 (Supporting Information). Therefore, the forward DNN is trained first to accurately predict spectral emissivity as a function of laser parameters (Figure 2a).

Once trained, the forward DNN, with fixed weights, serves as a differentiable surrogate model to train the inverse DNN, as shown in Figure 2b. Furthermore, the training loss for the inverse DNN is based directly on the difference between the input target emissivity and the optical properties predicted by the trained forward DNN for the laser parameters generated by the inverse DNN. This training loss is blind to whether or not the inverse DNN generates laser parameters that are different from the actual laser parameters in the training data corresponding to the input target emissivity under consideration. Consequently, the inverse DNN is free to discover and learn entirely different designs that may not even appear in the training data so long as they still produce the desired target optical properties. This training architecture therefore allows the inverse DNN to leverage the one‐to‐many mapping between optical properties and laser parameters.

The inverse DNN outputs 14 parameters, doubling as inputs for the forward DNN. These parameters comprise of continuous variables like scanning speed and spacing, with laser power represented as a discrete, one‐hot encoded variable. Emissivity values, spanning 800 evenly spaced wavelengths from 2.5 µm to 12 µm, serve as the inverse DNN's inputs and forward DNN's outputs. Both DNNs feature 16 hidden layers, as illustrated in Figure 2a,b. The scaled exponential linear unit (SELU) activation function is implemented in the initial linear layer and throughout all hidden residual layers within both the inverse and forward DNNs (Figure 2c). Each layer within the forward DNN encompasses 512 units, whereas the inverse DNN is structured with 1200 units per layer. Training was conducted for 350 epochs for the forward DNN, and extended to 2500 epochs for the inverse DNN, all within the TNN framework. Refer to the Methods section for a thorough overview of the hyperparameters and detailed inverse and forward DNNs architecture. The dataset for training both forward and inverse DNNs was randomly shuffled and split into training, validation, and testing segments with initial ratios of 80/10/10% (denoted as the 100% model). Also, in order to evaluate model robustness, 90% and 70% of the total 30380 samples were considered for training and validation, while the test set (3038 samples) was always the 10% of the whole dataset for control. The 90% and 70% models are denoted as data‐starved models and both used a 90/10% split for training and validation. The test versus predicted root mean squared error (RMSE) values for the 70%, 90% and 100% trained forward DNNs are 2.5%, 2.2%, and 2.3%, respectively (Figure 2d). Within the TNN framework, the training and validation loss of the inverse DNN converges to within 4% for all models (Figure 2e). Details on data preprocessing, validation strategy, and data starvation procedure are outlined in the Methods section.

## 4 Performance of the Trained TNN Model

**Figure 3:**
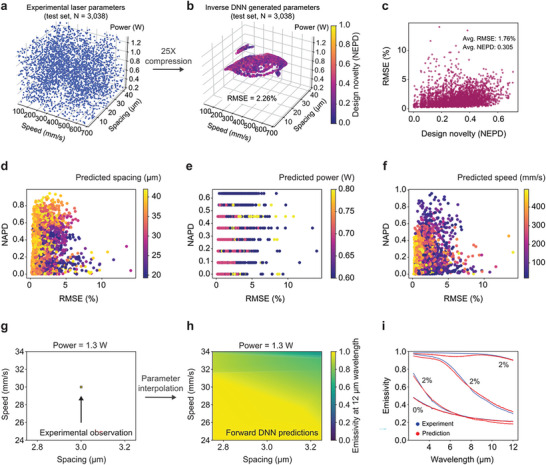
Performance validation for the 100% trained inverse and forward DNNs with the experimental test set. a) The experimental test set's laser parameters (number of test samples N = 3038). b) The inverse DNN‐generated laser parameters that map to the same emissivity curves as those in (a) comprise a compact region of laser parameter space 25 times smaller than the original test set in (a). c) Design novelty (NEPD) versus prediction error (RMSE) generated by the inverse DNN and validated by the forward DNN. NAPD results generated by the 100% inverse DNN for each laser parameter; d) spacing, e) power, and f) speed. g) Representative volume of laser parameter space corresponding to a single measurement (centered on 3.0 µm spacing, 30 mm s^−1^ speed, and 1.3 W of power) of emissivity at 12 µm wavelength, indicating sparsity of experimental data. h) Corresponding partial dependence plot for the same parameter region in (g) produced by the 100% trained forward DNN. i) Examples of the 100% forward DNN model predicted and experimental spectral emissivity. Corresponding RMSEs are listed as inset numbers.

**Figure** **3** shows performance metrics for both the forward DNN and the inverse DNN, trained utilizing the TNN framework. We employ two evaluative criteria: (*i*) RMSE (Equation 1) to assess the disparity in spectral emissivity between model‐predicted and experimentally‐observed emissivity curves, and (*ii*) normalized Euclidean parameters distance (NEPD, Equation 2) to quantify the normalized deviation between the inverse DNN‐generated laser parameters and those documented experimentally for the same optical properties. NEPD indicates the design novelty of generated laser parameters as compared to training data; 0 indicates identical laser parameters and 1 corresponds to the maximum possible difference between two sets of laser parameters (i.e., diagonally opposite corners of the 3D cube that is the full extent of the training data in laser parameter space). Figure 3a and Figure S7 (Supporting Information) show the test dataset (laser parameters and associated emissivity curves) used to evaluate the forward and inverse DNNs. These data are not part of the training dataset.

Figure 3b illustrates the laser parameters predicted by the 100% trained inverse DNN for the test set emissivities corresponding to the points in Figure 3a. Each parameter set is color‐coded based on its design novelty (NEPD) compared to Figure 3a. Figure S8 (Supporting Information) shows NEPD plots for all data‐starved inverse DNNs. Surprisingly, the volume of laser parameter space is reduced by a factor of 25, calculated as the ratio of the convex hull volumes enclosing the experimental and inverse DNN‐predicted parameters. The inverse DNN identifies a distinct, precise, and efficient prediction region in the parameter space, demonstrated by the clustering of accurately predicted parameters versus the test parameters. Interestingly, the design novelty (NEPD) is homogeneously distributed in this region, suggesting that when the model collapses distant laser parameter spaces into compact regions, it does not preserve the relative ordering of those points. Rather, this collapsed region is sufficiently expressive to reproduce all spectral emissivities (Figure S7, Supporting Information) with a high prediction accuracy (RMSE 2.26%). Small transitions within this subregion tend to induce large changes in corresponding optical properties. This reaffirms that the inverse DNN was effectively trained through the TNN approach (discussed in Figure 2) to utilize the complex many‐to‐one mapping governing this system. Moreover, parameter space compression can provide practical value for real applications by enabling the achievement of desired optical properties using hardware whose operation may be limited to specific regions within parameter space, such as due to manufacturing constraints like cost, throughput, or safety concerns. Additionally, this compression simplifies the inverse design problem, which could enhance design transferability and enable more optimized specialization of the manufacturing hardware.

Figure 3c shows the relationship between RMSE and NEPD, assessed by using the inverse DNN to predict laser parameters from test set emissivity curves and then feeding those predictions into the forward DNN to reconstruct predicted target emissivities, as illustrated in Figure 2b. The average RMSE of 1.76% confirms the model's inverse design accuracy for unobserved laser parameter designs. The average NEPD of 0.305 shows that in the majority of cases, the model biases towards designs considerably different from the training data. Rather than simply interpolating the training data, the model reproduces all optical properties by favoring clustered, compact volumes of the laser parameter space. The limited maximum NEPD (0.708) implies inherent physical limitations of the fabrication expressiveness; it may not always be possible to produce the target emissivity using arbitrarily distinct laser parameters (i.e., NEPD = 1). Figure 3d,e,f, and Figure S9 (Supporting Information) shows this degree of design novelty, as quantified by the normalized‐absolute‐parameter‐distance (NAPD), broken down by individual laser parameters generated by the inverse DNN and those observed experimentally, defined in Equation 3.

Figure 3g,h shows the forward DNN's continuous prediction of emissivity over the laser parameter space region near a single experimental observation. This demonstrates the model's ability to learn the complicated relationship between laser parameters and spectral emissivity, allowing for precise nonlinear interpolation. This is necessary to be able to explore and thus elucidate the functional relationships between laser parameters and resulting spectral emissivity. Neither brute force experiments nor numerical simulations are tractable approaches for generating a smooth plot such as in Figure 3g, thereby revealing design parameters worth further exploration with experiments or numerical simulations. Figures S10 –S12 (Supporting Information) provide more comprehensive visualizations of this learned emissivity function across the entire laser parameter space. Figure 3i displays representative spectral emissivity curves estimated by the 100% trained forward DNN with the laser parameters test set as input (Figure 3b). Each predicted curve is juxtaposed with its respective experimental counterpart, demonstrating a RMSE consistently measuring less than 3%.

## 5 Inverse Design of Photonic Surfaces via Inverse DNN for TPV Emitter

**Figure 4:**
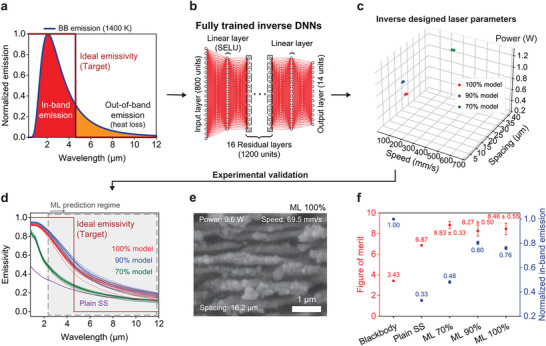
Experimental TPV emitter design. a) The ideal emissivity for a lead selenide‐based TPV emitter with a bandgap at 4.6 µm wavelength operating at 1400 K. b) The same fully trained inverse DNN, whose characteristics are shown in Figures 2 and 3, predicts laser parameters based on the target emissivity in (a). c) Inverse designed laser fabrication parameters using the 70%, 90%, and 100% models. d) Measured emissivities of substrates fabricated using laser parameters shown in (c). e) SEM image shows representative surface morphologies fabricated under laser parameters predicted by 100% model. The white scale bar is 1 µm. f) Figure of merits and in‐band emission normalized to blackbody for photonic surfaces.

Finally, we validate the efficacy of the inverse DNN experimentally using the spectral emissivity of a thermal emitter that could optimize the energy conversion efficiency of a TPV heat engine. TPVs convert thermal radiation into electricity using photovoltaics and have seen a recent surge in research leading to performances superior to some mature turbines.^[^ ^8b^ ^]^ Performance could be further enhanced by using a spectrally engineered thermal emitter. The ideal emitter would have an emissivity of 1 for wavelengths falling below the bandgap (referred to as in‐band emission) to co‐optimize heat‐to‐electricity conversion efficiency with power density. For wavelengths extending beyond the bandgap (out‐of‐band emission), the ideal emitter should have an emissivity of 0 to minimize losses. This ideal step‐shaped spectral emissivity is shown in **Figure** **4a** for a lead selenide‐based TPV that has a bandgap at 4.6 µm and converts heat at a temperature of 1400 K. This target emissivity also challenges our model as it is qualitatively different from all training data and it is not physically possible to perfectly achieve using SS.

The predicted designs for this target emissivity are fabricated onto SS plates using the laser parameters generated by the 100%, 90%, and 70% pretrained inverse DNNs (Figure 4b,c; specific laser parameters given in Table S2, Supporting Information) and their optical properties are measured using an FTIR spectrometer (Figure 4d). The nanostructured surface morphology of a representative TPV emitter fabricated using laser parameters predicted by the 100% inverse DNN is shown in Figure 4e. Collections of predictions (N = 14) were generated by perturbing the target ideal step function emissivity by 1%. Surprisingly, each model sourced predictions from separate compact regions of laser parameter space, and all models selected designs not seen during their training. The measured spectral range extends beyond the predictive scope of the TNN models and their training data, demonstrating potential for extrapolation. Notably, the spectral emissivity achieved through inverse design using the 100% and 90% DNNs closely resembles the ideal emissivity step function within the limits of what is physically achievable using textured SS. Compared to the intrinsic emissivity of pristine SS, the predicted designs’ emissivities are substantially elevated, from 0.49 to 0.93 at 0.8 µm wavelength for the 100% model (in‐band spectral range), while maintaining a low spectral emissivity at longer wavelengths (0.15 at 12 µm wavelength; out‐of‐band spectral range).

To evaluate the practical utility of the experimentally characterized thermal emitter designs generated by our model, we define a figure of merit (FOM) for spectrally engineered TPV thermal emitters. This FOM is calculated as the ratio of in‐band emissive power (desirable) to out‐of‐band emissive power (undesirable) that would be produced by the emitter during operation (Figure 4f; see derivations in Figure S13, Supporting Information). Higher values of the FOM are better. The baseline FOM value for a blackbody surface is 3.43. The FOMs from the measured properties of the emitters designed by the 70%, 90%, and 100% models are 8.83, 8.27, and 8.46, respectively, demonstrating significant enhancements. Furthermore, the in‐band emissive power normalized with respect to blackbody (theoretical maximum) demonstrates considerable enhancements of 145%, 242%, and 230% for the 70%, 90%, and 100% model, respectively, compared to plain SS. Notably, while the inverse DNN model trained on the 70% dataset produced similar validation RMSEs as the 90% and 100% models (Figure 2d,e; Figure S8, Supporting Information), it performed considerably worse than the 90% and 100% models for the real life TPV emitter design task considering in‐band emissive power (Figure 4d,f). This result indicates the 70% model's comparatively weaker ability to generalize beyond the training datasets, and highlights the importance of experimental validation beyond test data‐based validations. In contrast, the 90% model performs similarly to the 100% model, suggesting that not all the training data may be necessary. In summary, the experimentally fabricated model‐generated photonic surfaces significantly outperform both a blackbody and untextured SS on measures of energy conversion efficiency and power density, respectively. This experimental validation supports the ability of the inverse DNN to interpolate, extrapolate, and handle spectral emissivity targets qualitatively different from the training data.

## 6 Conclusion

The integration of high throughput fs laser fabrication techniques with a TNN framework has enabled the automatic inverse design of novel photonic surfaces. The experimentally validated trained TNN model utilizes the one‐to‐many mapping relationship between laser parameters and corresponding optical properties, generating novel designs not present within the training dataset and allowing the model to reproduce the full diversity of spectral emissivities using only a highly expressive compact region of laser parameter space 25 times smaller than required by the training data. While the current study focused on three laser parameters and one specific material, the approach can easily be generalized to encompass various materials, additional laser parameters (e.g., polarization, and repetition rate), and other possible constraints. This adaptability enhances its applicability across a range of energy harvesting and storage devices, including parabolic troughs, solar‐water desalination, and passive radiative cooling, where optimizing spectral emissivity and understanding its complicated functional dependence on device design details is essential for elevating overall system efficiency and performance. In the future, this approach could also be extended to other laser processing fields with complex relationships between laser parameters and material or process properties, such as laser‐based annealing and synthesis.^[^ ^17b,c^ ^]^

## 7 Experimental Section

### Materials

SS substrates (AISI 301, GoodfellowUSA) with 0.5 mm thickness were used as target specimens.

### High throughput fs Laser Fabrication

The fundamental 1030 nm wavelength of 500‐fs laser operating at a 100 kHz (s‐Pulse, Amplitude) repetition rate was focused via a galvano scanner (excelliSCAN 14, SCANLAB) with a beam spot size (30 µm in diameter), which allows the fabrication of different surface geometries under laser processing parameters on demand. A total of 35280 surfaces were fabricated using three different laser parameters (power, speed, and spacing) as shown in Figure S1 (Supporting Information), and each combination was made on 1 mm^2^ areas of SS substrates. A raster scanning method was used because this method was often employed in the field of fs laser fabrication technique.

### High throughput Optical Property Characterization

A custom FTIR spectrometer (Thermo Fisher Scientific, Nicolet iS50) microscope system was established for direct high throughput optical properties measurement of fabricated geometries (Figure S2, Supporting Information). The module was designed around an optical microscope configuration with a reflective objective lens (Thorlabs, LMM‐15X‐P01) and a liquid nitrogen cooled Mercury‐Cadmium‐Telluride detector to measure spectral reflectivity and corresponding emissivity from 2.5 µm to 12 µm wavelength. The system was synchronized with a set of motorized *XY* stages (KMTS50E, Thorlabs) for automated and high throughput measurements.

### DNN Architectures, Hyperparameters, Inputs and Outputs

The architecture of the forward DNN was composed of 16 hidden layers in total. Of these, two were linear layers, commonly referred to as fully‐connected or dense layers, while the remaining 16 were residual (linear) layers or blocks.^[^ ^22^ ^]^ Each of these hidden layers within the forward DNN comprises 512 units. The linear layers occupy the positions of the initial and final layers within the DNN structure, with the hidden residual layers inserted between them. The key feature of a residual layer was the introduction of a connection in which the original (untransformed) input of a layer was added to the (transformed) output of the same layer thereby mitigating the vanishing gradient problem which was usually specific to deeper networks like the one used in this study. The structure of the residual layers used within the DNNs was presented in Figure 3c. Last, the sigmoid activation function was applied at the output layer since all the emissivity values across the 800 wavelengths range from 0 to 1. In contrast, the inverse DNN mirrors the forward DNN in terms of the quantity and sequence of hidden layers. However, it differs in the unit count, hosting 1200 units within both its linear and residual layers. The final linear layer of the inverse DNN maps the 14 output parameters without an activation function. The laser power variable was handled using a one‐hot encoding technique, representing it as 12 distinct values for each power level. In this schema, the active power level was marked as 1, with all others set to 0. Considering this, and the two continuous variables–scanning speed and spacing, a total of 14 variables were used as inputs for the forward DNN, and as outputs for the inverse DNN.

The SELU activation function was uniformly applied across both DNNs.^[^ ^23^ ^]^ Specifically, it was utilized in the initial linear layer and all the residual layers in both the forward and inverse DNNs. Among the various hyperparameters configured, both the forward and inverse DNNs utilized a batch size of 4, a gamma value of 0.1 for learning rate decay, and a weight decay of 0.01. The initial learning rates were set to 10^−4^ for the forward DNN and 10^−6^ for the inverse DNN. As for the training duration, the forward DNN was trained for 350 epochs, while the inverse DNN underwent 2500 epochs of training. The AdamW optimizer was employed for optimizing both networks. The DNN architectures and hyperparameters were determined through computational experimentation and a hyperparameter grid‐search process. The deep learning module PyTorch 1.11.0 for Python 3.10 was used to build and train the DNN models.^[^ ^24^ ^]^

### Dataset Preprocessing and TNN Validation Strategy

Of the 35280 data samples obtained through experimental procedures, 30380 samples were earmarked for training, validating, and testing the TNN model. The reason for this selective inclusion lies in maintaining the consistency of the emissivity curves across the wavelength domain. A subset of the data have to be excluded because it requires minor extrapolation to align with the rest, due to inconsistencies in the measurements. By discarding this portion, it was ensured that the dataset retained its uniformity, thereby improving the model's potential for accurate and reliable performance.

The dataset underwent random shuffling and was partitioned into an 80/10/10% split for the training, validation, and testing phases, applicable to both the inverse and forward DNNs. Additionally, a sensitivity and robustness analysis was conducted on the models. In this procedure, referred to as “data starvation,” the volume of data allocated for training and validation (80/10) was systematically scaled down. Specifically, the DNNs were trained using the full dataset for training and validation, as well as reduced subsets containing 90% and 70% of the original data.

### Model Analysis

The RMSE and the NEPD were used to assess the performance of the ML model in terms of accuracy and laser parameters design novelty, and were defined in Equation 1 and Equation 2, respectively.

The RMSE was selected as the metric for evaluating the discrepancy between the model‐predicted and experimentally‐observed emissivity curves, primarily because of its intuitive interpretability.

(1) $$RMSE\;=\frac{100}{\varepsilon_{max}-\varepsilon_{min}}\;\sqrt{\frac{1}{N\times M}\sum_{i\;=\;1}^{N}\sum_{j\;=\;1}^{M}{\left(\varepsilon_{i,j}^{T}-\varepsilon_{i,j}^{P}\right)}^{2}}$$

In Equation 1, *N* represents the total number of test samples, while *M* signifies the number of distinct wavelengths at which emissivity was measured for each test sample. In the given context, each sample *i* includes 800 emissivity values corresponding to 800 different wavelengths. *T* and *P* stand for the true (experimental) and predicted emissivity values *ϵ*, respectively, at a specific wavelength *j*. The *mean_squared_error* function, available in version 1.2.2 of the scikit‐learn module for Python 3.10,^[^ ^25^ ^]^ was utilized to compute the RMSE value. Although this function intrinsically calculates the mean squared error, the square root of the obtained value was further computed to derive the RMSE. Subsequently, the RMSE was normalized by the range of maximum and minimum theoretical emissivity (ε_*max*_, ε_*min*_) values, which was equal to 1, and then multiplied by 100 to express the relative RMSE value as a percentage.

To assess the design novelty of each predicted parameter as the NEPD metric was introduced through the following equation:

(2) $$\;NEPD_{i}=\frac{1}{\sqrt{3}}\;\times \sqrt{\sum_{k\;=\;1}^{3}{\left(L_{i,k}^{Tn}-L_{i,k}^{Pn}\right)}^{2}}$$

where 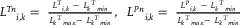


*L^Tn^*_*i*, *k*_ denotes the normalized *k*‐th parameter of the *i*‐th true test sample while *L^Pn^*_*i*, *k*_ was the normalized *k*‐th parameter of the predicted test sample. The value *L*_*i*,*k*_^*T*^ denotes the *k*‐th parameter of the *i*‐th true test sample, while *L*_*i*,*k*_^*P*^ was the *k*‐th parameter of the *i*‐th predicted instance, whereas *L_k_* ^*T*^_*max*_ and *L_k_* ^*T*^_*min*_ were each of the *k* parameters maximum and minimum values. The parameter index *k* takes values 1, 2, or 3, indicating the three distinct laser manufacturing parameters being considered for each instance *i*.

To ascertain the inverse DNN‐predicted laser parameter set compression factor in juxtaposition with experimental test set parameter values, the volume of the convex hull was computed within both the predicted and actual parameter spaces. The compression factor, denoted as *c*, was formulated through the expression *c*  =  *V_ch_* ^*exp*^ / *V_ch_* ^*dnn*^, where *V_ch_* ^*exp*^ represents the convex hull volume derived from the experimental test set, whereas, *V_ch_* ^*dnn*^ illustrates the convex hull volume emanating from the inverse DNN‐predicted laser parameters. The normalized parameters, as per Equation 2, were used to calculate the volume and the compression factor. The ConvexHull function within scipy's 1.11.1 spatial module for spatial algorithms as a part of Python 3.10 was used for convex hull volume calculation.^[^ ^26^ ^]^

NAPD was employed to quantify the difference between individual laser parameters generated by the inverse DNN and those observed experimentally, defined in Equation 3.

(3)

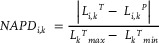




### Data Availability

The data supporting the findings of this study were available within this article and its Supporting Information files. Source data were available in https://osf.io/79pa4/.

## Conflict of Interest

The authors declare no conflict of interest.

## Supporting information

Supporting Information

Supplementary Video S1

## Data Availability

The data that support the findings of this study are available from the corresponding author upon reasonable request.
