# Supplementary material for: Inverse Design of Photonic Surfaces via High throughput Femtosecond Laser Processing and Tandem Neural Networks
Source: Adv Sci (Weinh). 2024 Apr 29;11(26):2401951. doi: 10.1002/advs.202401951 (PMC11234413; doi:10.1002/advs.202401951)
Supplement: Supplementary file 1 — Supporting Information [file ADVS-11-2401951-s001.pdf]

ADVANCED SCIENCE
Open Access

Supporting Information

for *Adv. Sci.*, DOI 10.1002/advs.202401951

Inverse Design of Photonic Surfaces via High throughput Femtosecond Laser Processing and Tandem Neural Networks

Minok Park, Luka Grbčić, Parham Motameni, Spencer Song, Alok Singh, Dante Malagrino, Mahmoud Elzouka, Puya H. Vahabi, Alberto Todeschini, Wibe Albert de Jong, Ravi Prasher, Vassilia Zorba* and Sean D. Lubner**

Supporting Information

Inverse design of photonic surfaces via high throughput femtosecond laser processing and tandem neural networks

Minok Park, Luka Grbčić, Parham Motameni, Spencer Song, Alok Singh, Dante Malagrino, Mahmoud Elzouka, Puya H. Vahabi, Alberto Todeschini, Wibe Albert de Jong, Ravi Prasher, Vassilia Zorba*, and Sean D. Lubner**

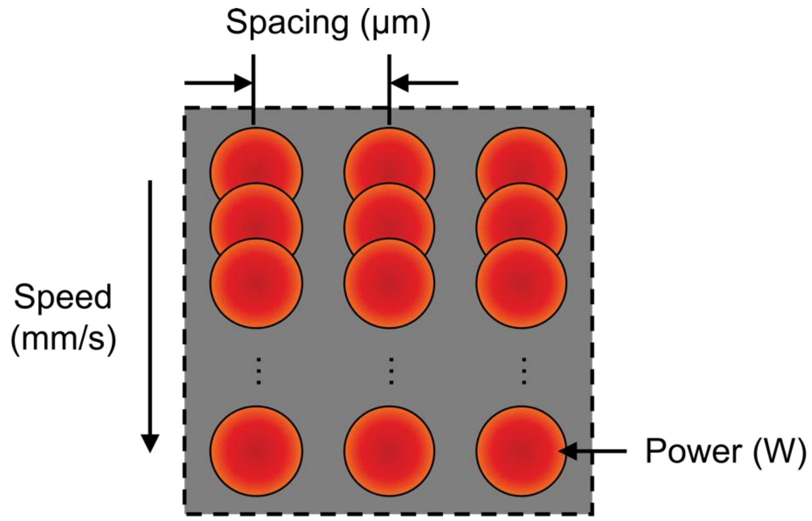

	Variables					Increment
Spacing (μm)	1	2	3	···	42	1 (μm)
Speed (mm/s)	10	20	30	···	700	10 (mm/s)
Power (W)	0.2	0.3	0.4	···	1.3	0.1 (W)

Figure S1. Schematic of ultrafast fs laser processing with a raster scanning method. Three different laser parameters including laser power, scanning speed, and spacing are used to texture the target surface area. Using variables as parameter inputs, a total of 35,280 different surfaces were generated.

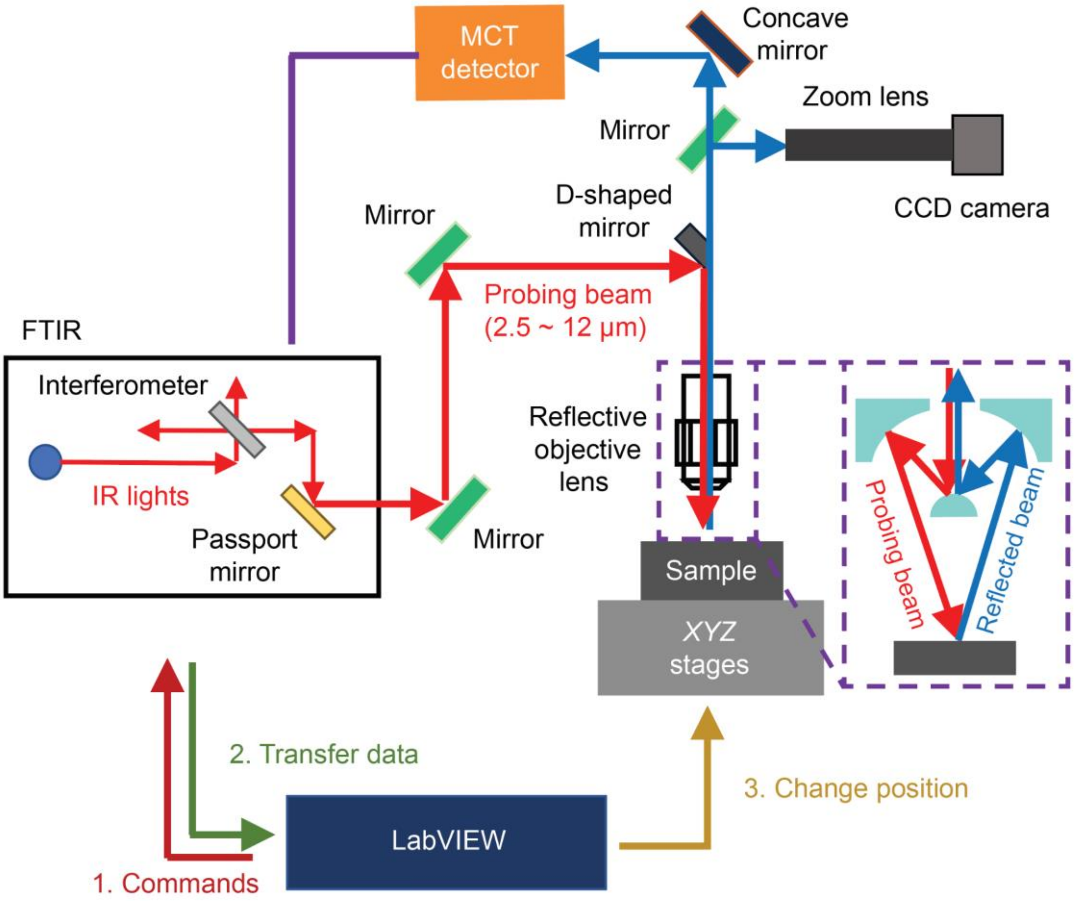

Figure S2. Schematic of the custom Fourier Transform Infrared spectrometer (FTIR) microscope system. A reflective objective lens was used to focus the IR beam on the target surface. To achieve high signal to noise ratios for acquired data, a liquid-nitrogen cooled Mercury-Cadmium-Telluride detector was coupled with the FTIR. LabVIEW software was used to synchronize the FTIR equipment and motorized stages for automated high throughput optical property measurements.

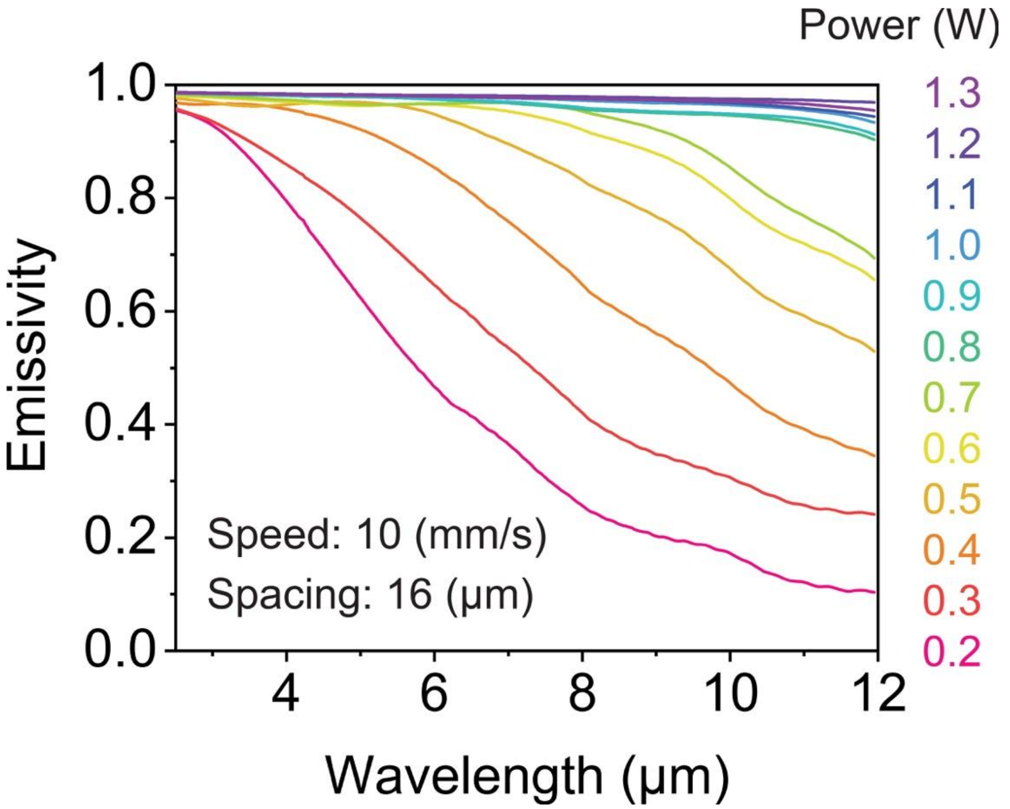

Figure S3. Examples of spectral emissivity of photonic structures laser-fabricated on stainless steel by changing the laser power while maintaining the same spacing of 16 μm and speed of 10 mm/s.

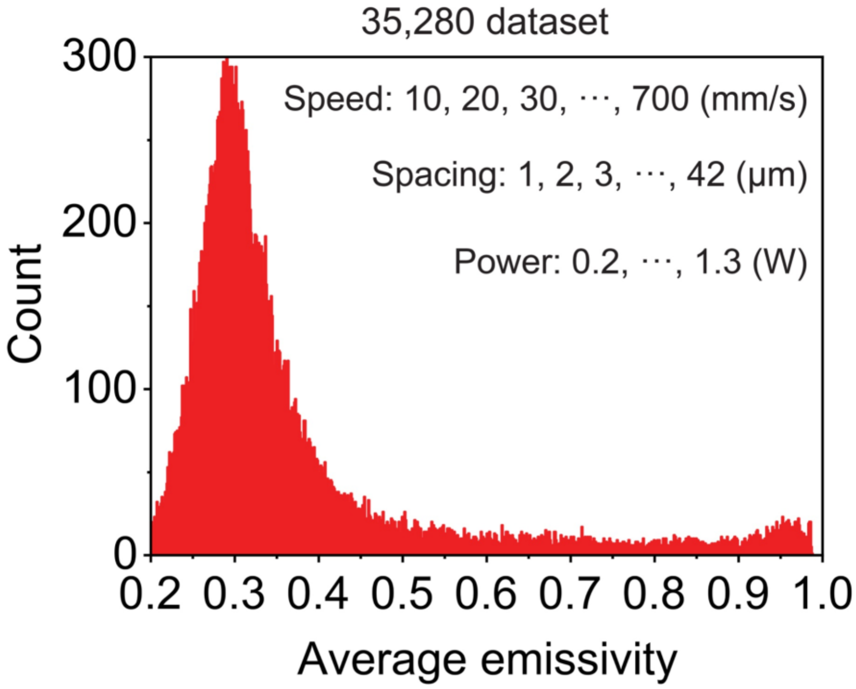

Figure S4. Average emissivity of 35,280 photonic surfaces fabricated on stainless steel under different laser processing conditions.

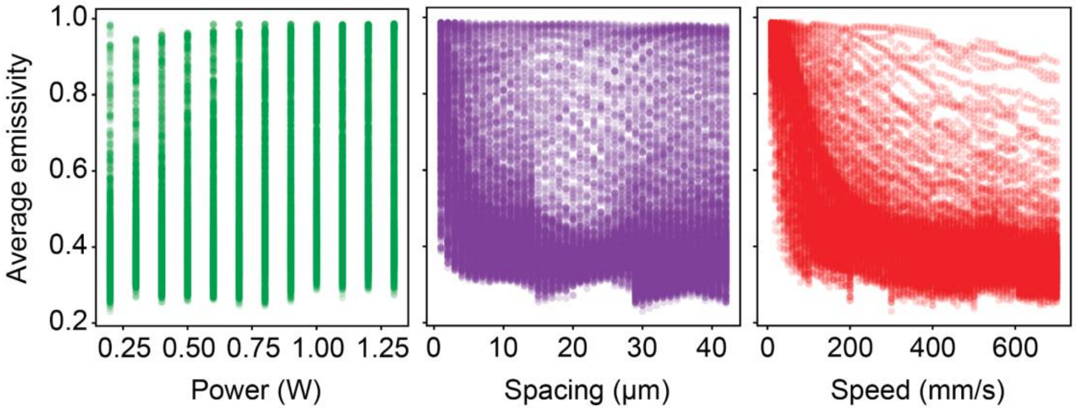

Figure S5. Average emissivity distribution of 35,280 photonic surfaces as a function of power, spacing, and speed, respectively.

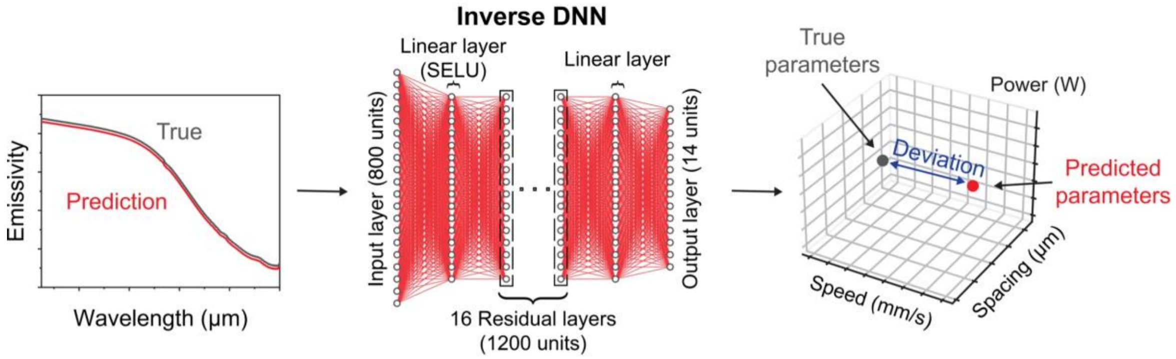

Figure S6. The schematic representation of one-to-many mapping in inverse design.

In the context of training an inverse deep neural network (DNN), challenges arise when it is not incorporated within the tandem neural network (TNN) framework. These challenges stem primarily from the problem's inherent one-to-many mapping nature. While the objective during DNN training is to minimize the loss function and fine-tune the weights, an intriguing observation emerges; even if the predicted emissivity (highlighted in red) closely aligns with the true emissivity, there may remain a significant difference between the true and predicted parameters. This disparity is largely due to the one-to-many relationships involved. Incorporating a trained forward DNN into the TNN framework addresses this challenge. By using an outer loss function to compare the true and predicted emissivity curves, the inverse DNN can be more accurately calibrated.

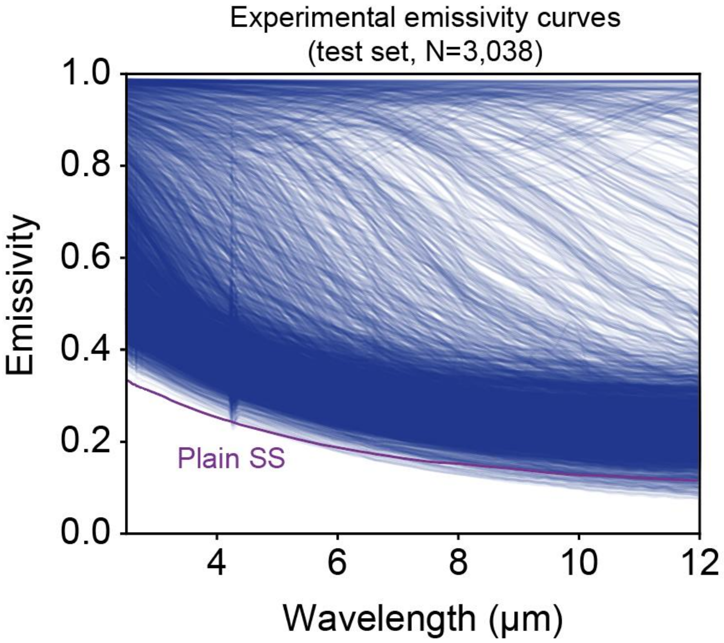

Figure S7. The experimental emissivity curves (N = 3,038) that correspond to the test set laser parameters presented in **Fig. 3b**.

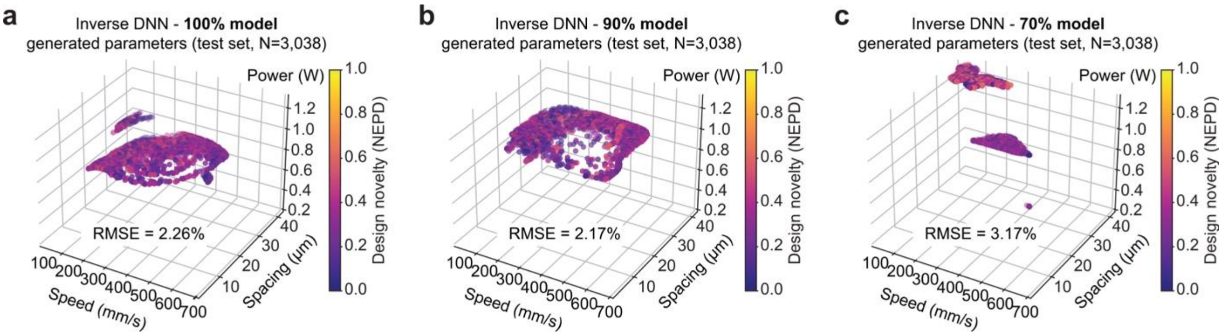

Figure S8. Test set laser parameters generated by the inverse DNN, colored by the Normalized Euclidean Parameters Distance (NEPD) to indicate design novelty; (a) 100% model, (b) 90% model, and (c) 70% model, respectively.

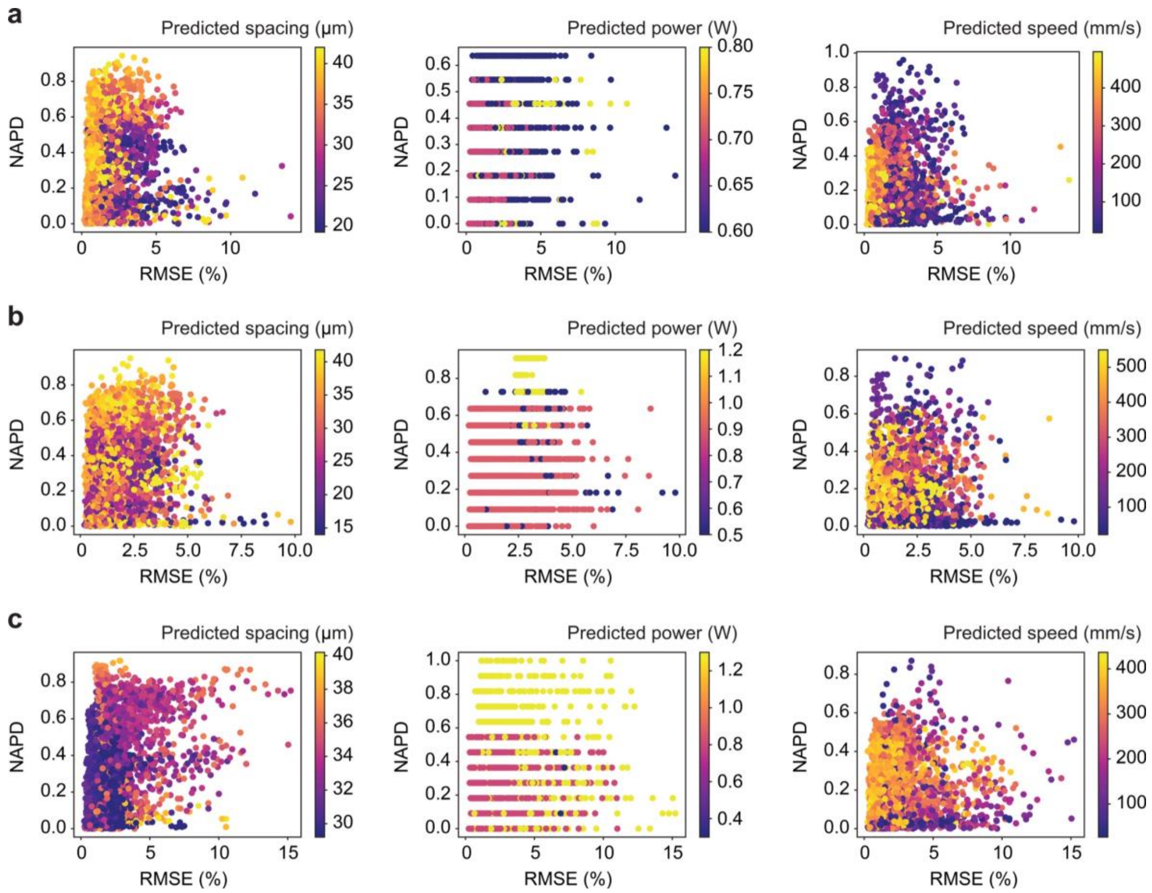

Figure S9. Normalized Absolute Parameter Distance (NAPD) results using Equation S1 generated by the inverse DNN for each laser parameter separately; (a) 100% model, (b) 90% model, and (c) 70% model, respectively.

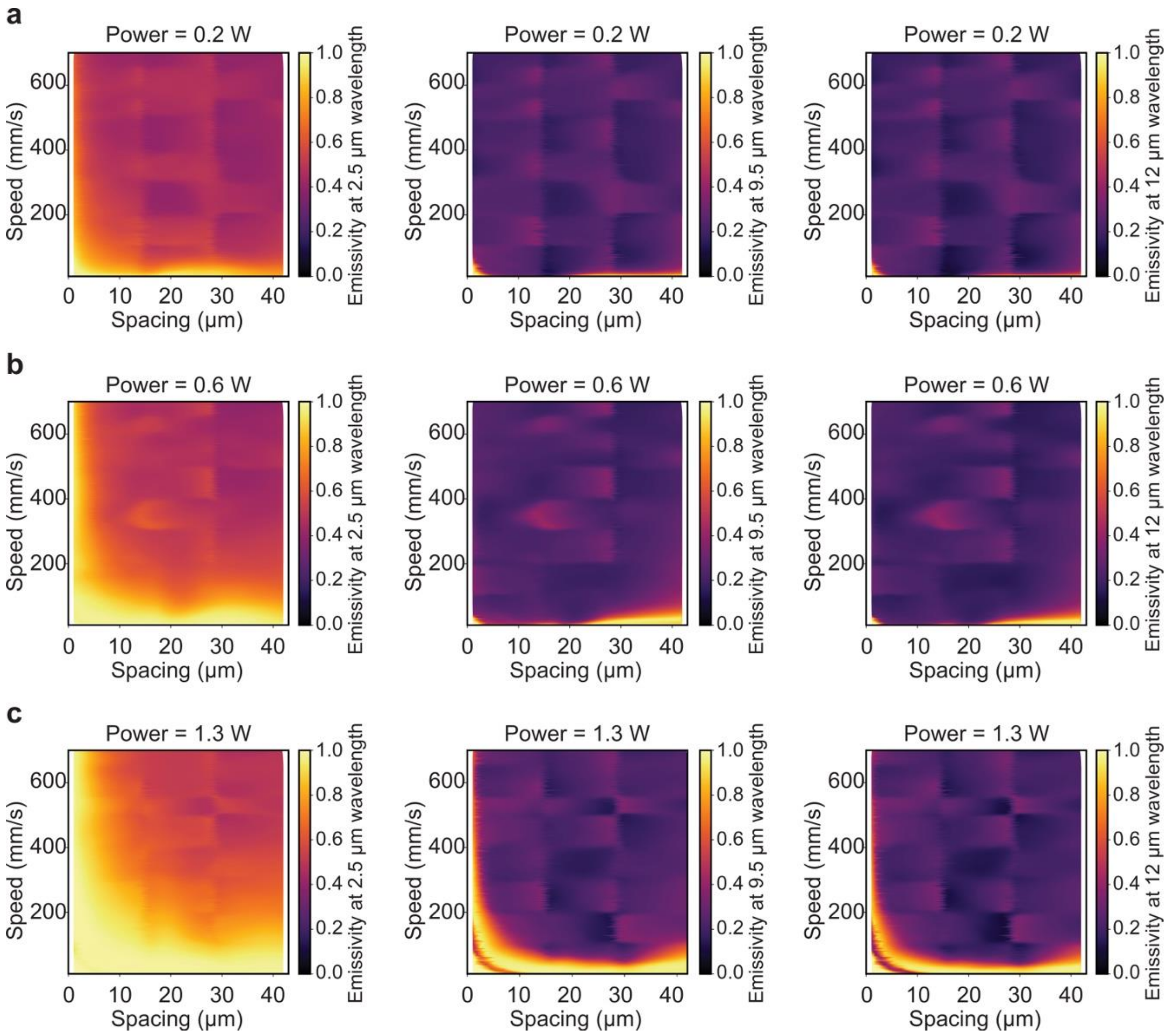

Figure S10. Partial dependence plot of spectral emissivity with respect to the speed and the spacing at the fixed power of (a) 0.2 W, (b) 0.6 W, and (c) 1.3 W, respectively.

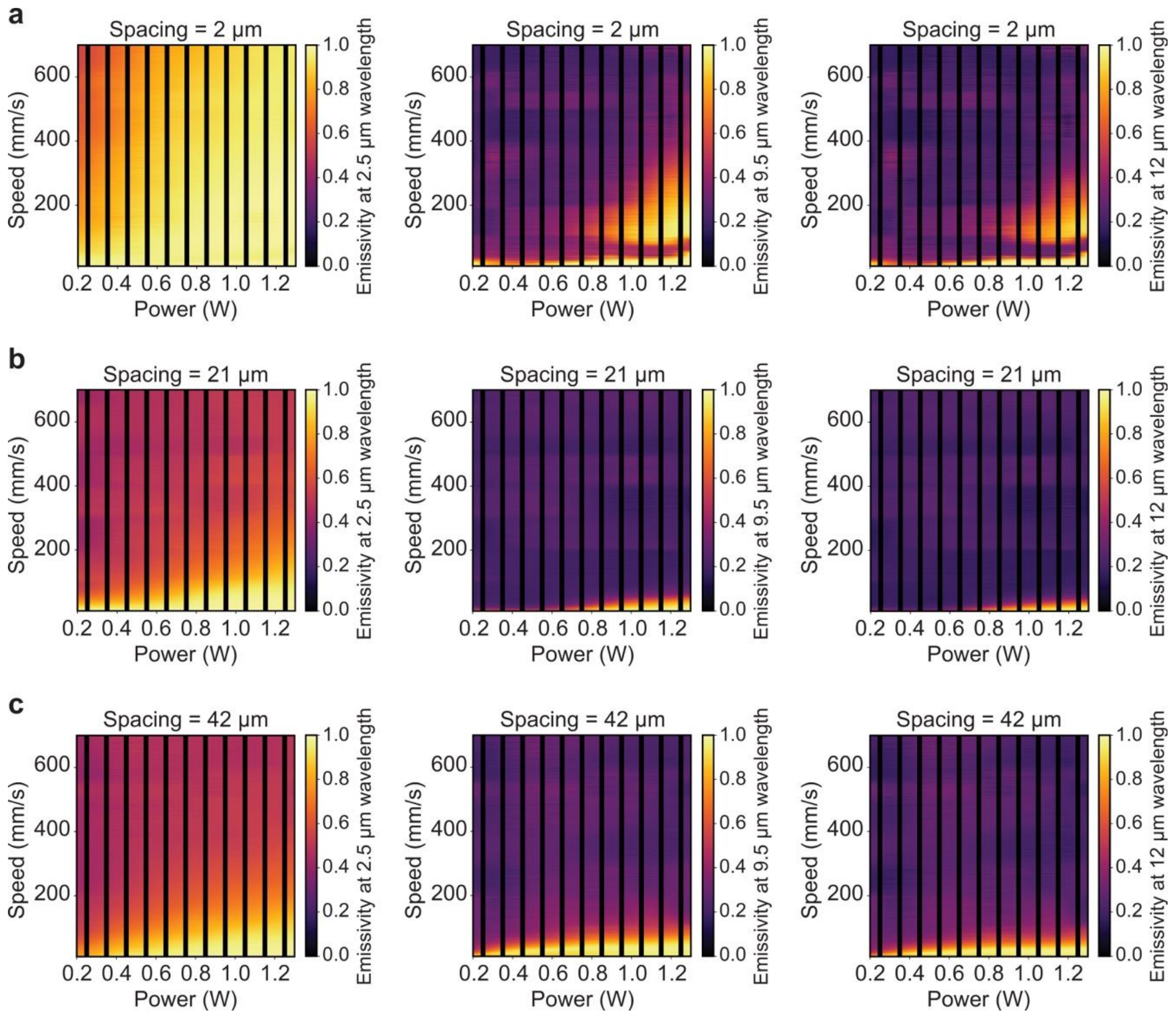

Figure S11. Partial dependence plot of spectral emissivity with respect to the speed and the power at the fixed spacing of (a) 2 μm, (b) 21 μm, and (c) 42 μm, respectively.

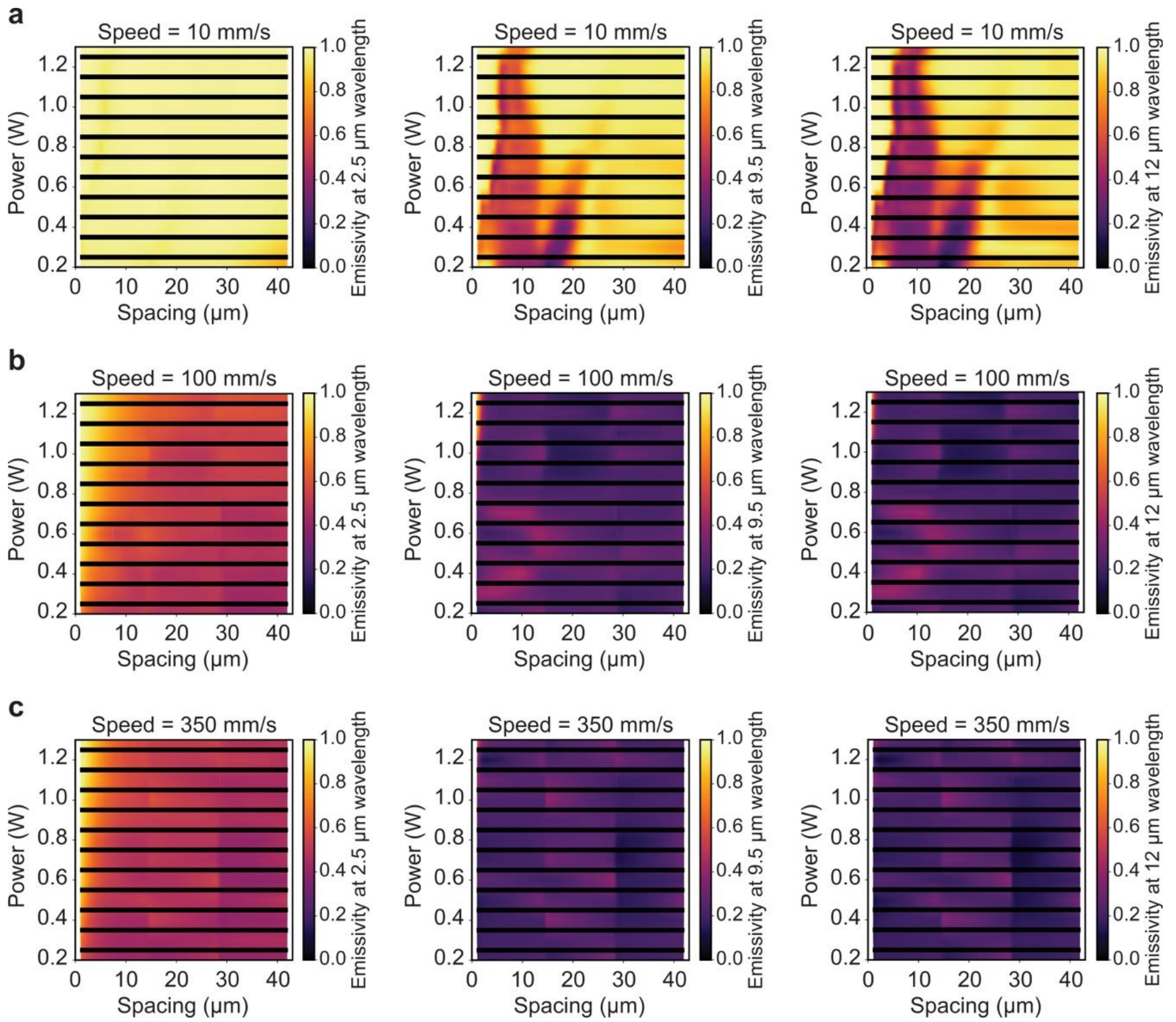

Figure S12. Partial dependence plot of spectral emissivity with respect to the power and the spacing at the fixed speed of (a) 10 mm/s, (b) 100 mm/s, and (c) 350 mm/s, respectively.

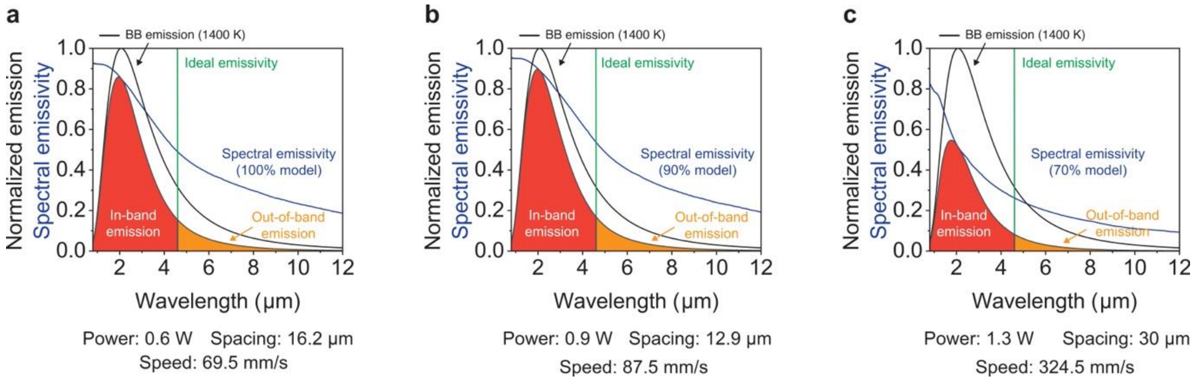

Figure S13. Weighted thermal emission and spectral emissivity for (a) 100% model, (b) 90% model, and (c) 70% model, respectively. Representative laser parameters for each model are listed as inset numbers.

The emission powers and associated figure of merits (FOMs) are calculated by integrating the emissivity, weighted by a Plank distribution at 1400 K, from zero microns up to the bandgap for in-band emission, and from the bandgap out to 12 μm wavelength for the out-of-band emissions. Specifically, weighted thermal emission, $W\ (\lambda, T)$, can be calculated based on Planck's blackbody radiation, as expressed below.

$$W\ (\lambda, T)\ = \varepsilon(\lambda)\ \times\ \frac{2hc^2}{\lambda^5}\frac{1}{e^{hc/(\lambda k_B T)}-1}$$

where, $\varepsilon(\lambda)$ is the spectral emissivity, h is the Planck's constant, c is the speed of light, λ is the wavelength, k_B is the Boltzmann constant, and T is the temperature of 1400 K.

Power (W)	0.2	0.3	0.4	0.5	0.6	0.7	0.8	0.9	1.0	1.1	1.2	1.3
Intensity (W/cm^2)	0.28×10^5	0.42×10^5	0.57×10^5	0.70×10^5	0.85×10^5	0.99×10^5	1.13×10^5	1.27×10^5	1.41×10^5	1.56×10^5	1.70×10^5	1.84×10^5
Fluence (J/cm^2)	0.28	0.42	0.57	0.70	0.85	0.99	1.13	1.27	1.41	1.56	1.70	1.84

Table S1. Laser processing parameters in power (W), intensity (W/cm^2), and pulse fluence (J/cm^2) at a 100 kHz repetition rate.

Sample number	100% model			90% model			70% model		
	Power (W)	Spacing (μm)	Speed (mm/s)	Power (W)	Spacing (μm)	Speed (mm/s)	Power (W)	Spacing (μm)	Speed (mm/s)
1	0.6	16.2	69.5	0.9	12.6	95.8	0.7	29.7	352.1
2	0.6	16.2	69.8	0.9	11.8	93.1	1.3	30	324.5
3	0.6	16	72.9	0.9	12.5	91.5	1.3	30.3	343.7
4	0.6	16.2	74	0.9	12.1	98.1	1.3	29.5	346.8
5	0.6	16.1	73.2	0.9	12.9	87.5	1.3	29.5	347.8
6	0.6	16.2	72.2	0.9	12.6	95.9	0.7	29.5	358.5
7	0.6	15.9	73.1	0.9	12.2	91.8	1.3	29.1	328.2
8	0.6	16.8	70	0.9	11.5	93.9	1.3	29	332
9	0.6	16	71.8	0.9	12	92.7	1.3	29.4	348.6
10	0.6	16.7	73.1	0.9	11.6	91.9	1.3	29.4	348.6
11	0.6	16	72.2	0.9	12.6	90.3	1.3	29.8	334
12	0.6	16.7	73.1	0.9	12.7	97.7	1.3	29.8	327.2
13	0.6	16.6	81.8	0.9	12	91.8	1.3	29.9	343.5
14	0.6	15.5	69.6	0.9	12.1	96.1	1.3	30.1	335.3

Table S2. Laser processing parameters predicted by the trained Inverse DNN models for the TPV ideal emissivity target shown in **Fig. 4d**.

Video S1. High throughput femtosecond laser processing to fabricate photonic surfaces
